# Case Report: Organ procurement in a DCD donor with ovarian thecoma: abdominal NRP enabled timely and safe resection, pathological confirmation, and successful kidney transplantation

**DOI:** 10.3389/frtra.2025.1680491

**Published:** 2025-10-02

**Authors:** Benjamin Assouline, Timothée Olivier, Anne-Laure Rougemont, Philippe Compagnon, Charles-Henri Wassmer, Hervé Quintard, Karim Bendjelid, Franz Immer, Raphaël Giraud

**Affiliations:** ^1^ Intensive Care Division, Department of Acute Care Medicine, Geneva University Hospitals, Geneva, Switzerland; ^2^ Department of Anesthesiology, Pharmacology, Intensive Care and Emergency Medicine, Faculty of Medicine, University of Geneva, Geneva, Switzerland; ^3^ Geneva Hemodynamic Research Group, Faculty of Medicine, University of Geneva, Geneva, Switzerland; ^4^ Division of Oncology, Geneva University Hospitals, Geneva, Switzerland; ^5^ Division of Clinical Pathology, Geneva University Hospitals, Geneva, Switzerland; ^6^ Division of Transplantation, Geneva University Hospitals, Geneva, Switzerland; ^7^ Swisstransplant, Bern, Switzerland

**Keywords:** normothermic regional perfusion, pathological diagnosis, organ procurement, organ transplantation, ovarian serous cystadenofibroma, thecoma

## Abstract

**Background:**

Donation after circulatory death (DCD) may be complicated by incidental findings, including tumor lesions that require urgent diagnosis. Here, we describe the case of a DCD donor with a large adnexal mass. Abdominal normothermic regional perfusion (A-NRP) enabled the safe resection of the mass, real-time pathological analysis, and subsequent kidney transplantation.

**Case summary:**

A 60-year-old woman suffered a hypoxic cardiac arrest and subsequently remained in a deep coma with poor neurological prognostic indicators. In accordance with her presumed wishes, life support was withdrawn, and a controlled DCD procedure with A-NRP was initiated. Imaging revealed a 27-cm adnexal mass. Laboratory markers showed elevated cancer antigen 125 (CA 125) but low cancer antigen 19-9 (CA 19-9) and carcinoembryonic antigen (CEA), and cytology was negative. Bilateral oophorectomy was performed under A-NRP, and the frozen section excluded malignancy, with final pathology confirming an ovarian thecoma. Both kidneys were procured; only the left kidney was transplanted successfully. The recipient experienced immediate diuresis and regained stable renal function at 1 month.

**Discussion:**

This case illustrates how A-NRP provides oxygenated perfusion while allowing time for surgical excision and a pathological diagnosis of incidental tumors. It prevented unnecessary donor exclusion and enabled transplantation.

**Conclusion:**

In selected DCD donors with incidental lesions, A-NRP can safely bridge the diagnostic process, preserve organ viability, and expand the donor pool.

## Introduction

Worldwide, organ donation is constrained by organ availability. In Switzerland, patients awaiting transplantation greatly outnumber donors (1), and nearly 100 individuals die annually while on the waiting list. Thus, high detection rates of potential donors and minimal exclusions are crucial. Organ evaluation is a comprehensive exercise involving blood tests, imaging, and screening for contraindications, notably malignancy. However, cancer is no longer an absolute contraindication unless it is metastatic. Incidental findings of unknown masses on CT scans are common and often delay organ donation, adding stress to families.

In 2023, donation after circulatory death (DCD) accounted for approximately half of organ donations in Switzerland (2). Two procurement methods are employed: super rapid recovery (SRR, or crush laparotomy) and abdominal normothermic regional perfusion (A-NRP). Compared with SRR, A-NRP enables *in situ* reperfusion and is associated with better outcomes in kidney and liver transplantation (3). In Switzerland, controlled DCD (Maastricht category III) is the standard practice. According to the 2023 national report, there were 200 deceased donors, with 104 donation after brain death (DBD), and 96 DCD, accounting for 48% of all donations.

Here, we report a DCD case involving a large abdominal mass. Following a multidisciplinary discussion (with the ICU team, the Swiss National Transplant Society, the donation team, and the oncology team), A-NRP was used to maintain organ perfusion during mass excision and histology, ultimately allowing kidney procurement.

## Case description

**Figure 1:**
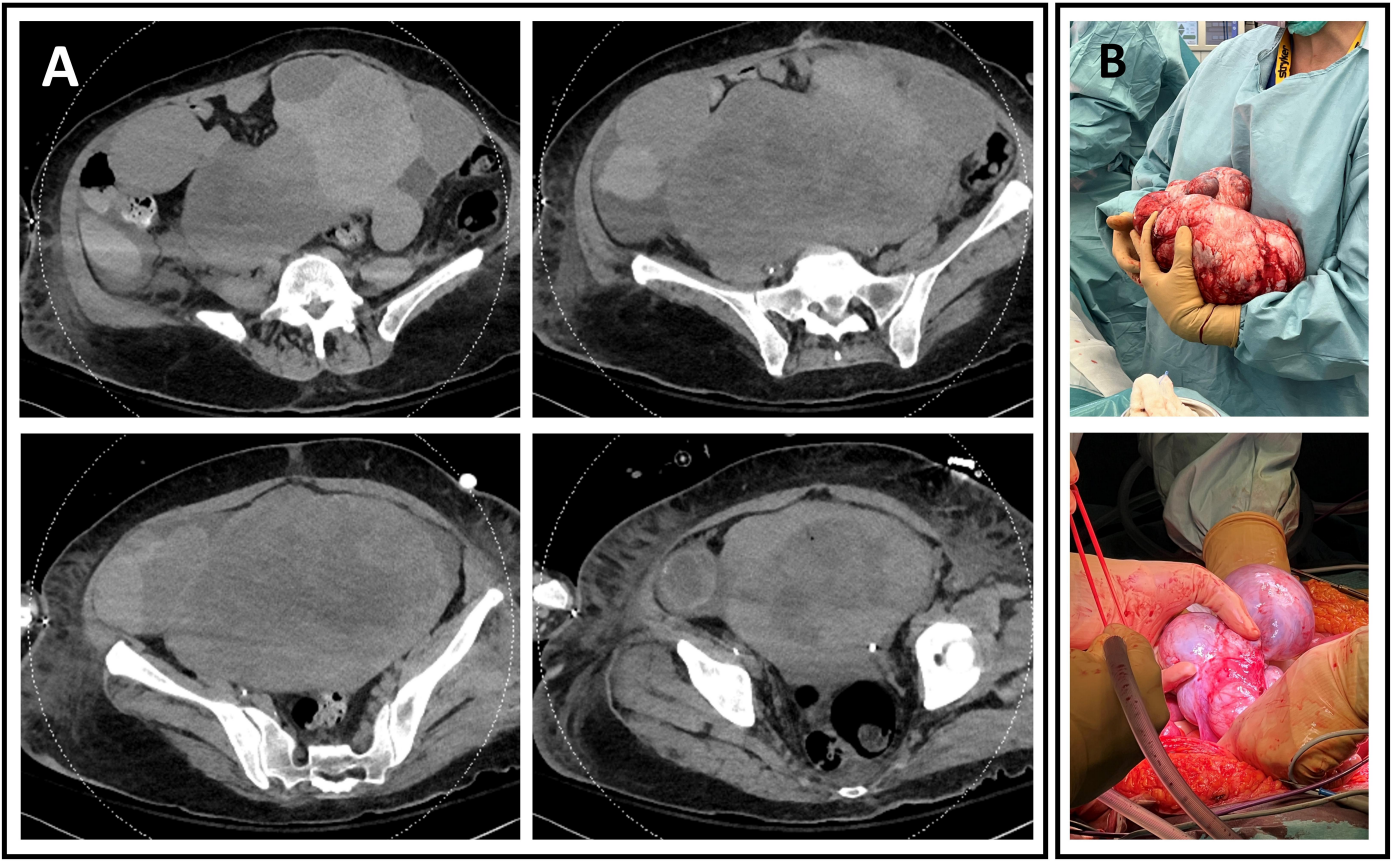
(**A**) Abdominal CT scan showing a large, heterogeneous, fleshy, and hypervascularized cystic mass measuring 27.2 cm wide, 14.1 cm deep, and 21.7 cm high. (**B**) Perioperative excision of two large multiloculated cystic masses. (Top) Left mass, weighing 2,932 g, measuring 27 cm × 20 cm × 20 cm. (Bottom) Right mass, weighing 436 g and measuring 15 cm × 8 cm × 5 cm.

A 60-year-old woman was admitted to our ICU after a cardiac arrest. Her history was largely unknown aside from active smoking. According to her husband, she had progressive dyspnea for several weeks but sought no medical advice. One night, she coughed up blood and collapsed. Her husband initiated CPR; paramedics arrived 15 min later and provided advanced life support. Return of spontaneous circulation (ROSC) was achieved after 10 min. The initial rhythm was asystole, with a no-flow time of 15 min and a low-flow time of 10 min. She was intubated, ventilated, and stabilized with epinephrine. An electrocardiogram showed sinus tachycardia without signs of acute coronary syndrome. A CT scan ruled out intracranial hemorrhage and pulmonary embolism, but it revealed a large necrotic mass near the adnexa with ascites (Figure 1), hypodense liver lesions, and bilateral pleural effusions with lower lobe atelectasis.

Sedation was stopped early, but the patient remained comatose even after 72 h. Neurological prognostication revealed a poor prognosis: absent brainstem reflexes (corneal, oculocephalic, and cough), a highly malignant EEG, absent somatosensory evoked potentials, elevated neuron-specific enolase (162.8 µg/L), and diffuse anoxic lesions on brain MRI.

**Figure 2:**
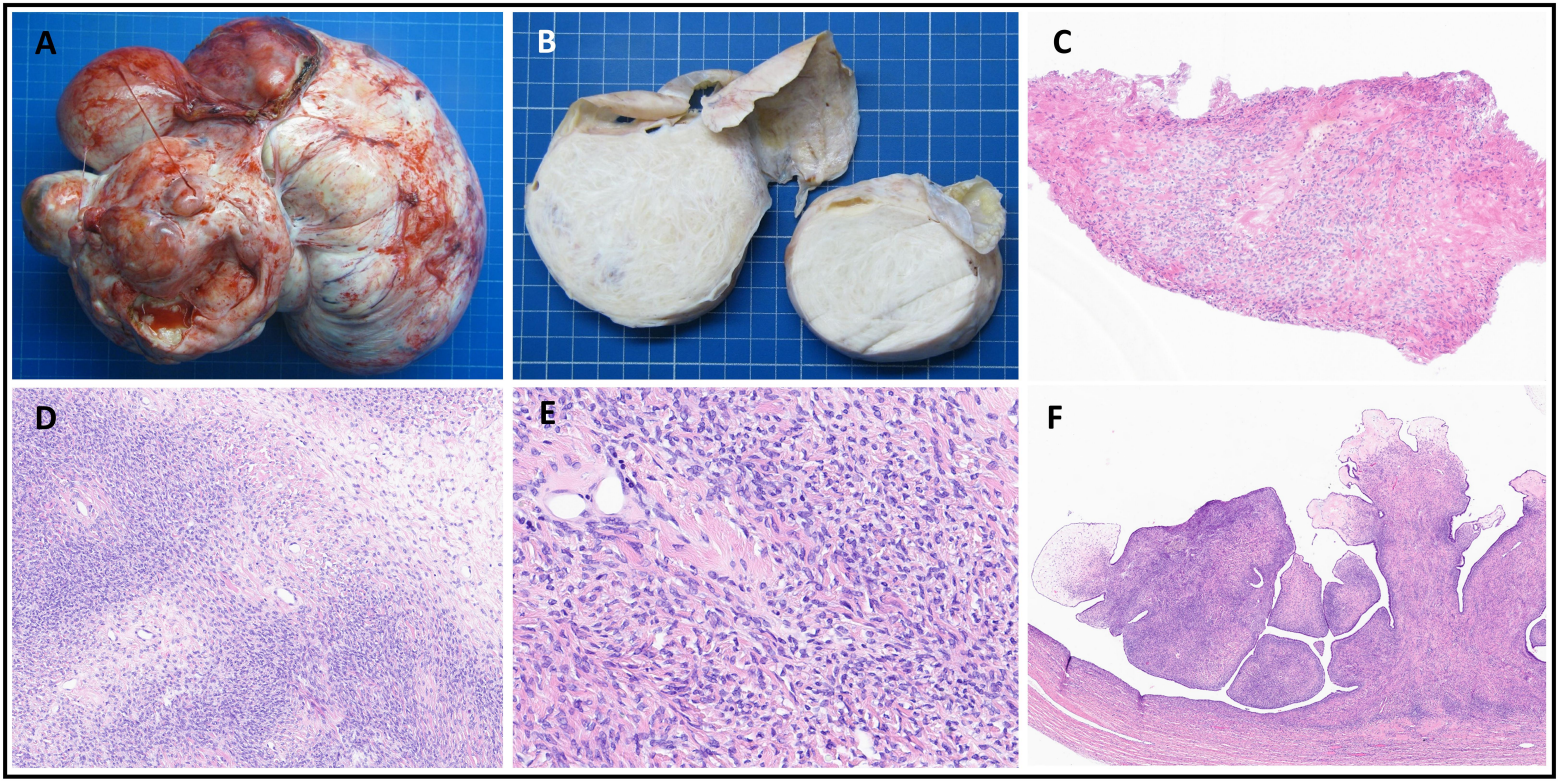
Pathology findings. (**A,B**) Gross findings of the left ovarian mass: a multiloculated cystic tumor (measuring 27 cm × 20 cm × 20 cm) with well-circumscribed, pale yellow fascicular areas. (**C**) Frozen section showing spindle cell proliferation without atypia (H&E, ×100). (**D,E**) Histology confirming ovarian thecoma with uniform spindle cells and collagen deposition (H&E, ×100–240). (**F**) Serous cystadenofibroma with fibromatous papillae lined by a Fallopian tube–like epithelium (H&E, ×240).

With family consent, therapeutic withdrawal was planned. Organ donation was considered, despite the adnexal mass. The levels of cancer antigen 19-9 (CA 19-9), cancer antigen 125 (CA 125), and carcinoembryonic antigen (CEA) were 10 kU/L, 537 kU/L, and 1.6 μg/L, respectively. The cytology of the pleural and ascitic fluids showed no malignancy. A percutaneous biopsy under ultrasound guidance revealed spindle cell proliferation in fibrocollagenous stroma without significant atypia (Figure 2). The liver was deemed unsuitable for transplantation due to hepatomegaly and heterogeneous hepatic lesions on imaging, raising concerns of an underlying pathology. A DCD heart was not offered, as DCD heart retrieval under A-NRP was not being performed in our center at that time. The lungs were not accepted for transplantation due to pneumonia, radiological infiltrates, and poor gas exchange on blood gases. Kidney function was preserved, and both kidneys were proposed for transplantation; however, acceptance was contingent upon prior resection and histological confirmation of the mass.

**Figure 3:**
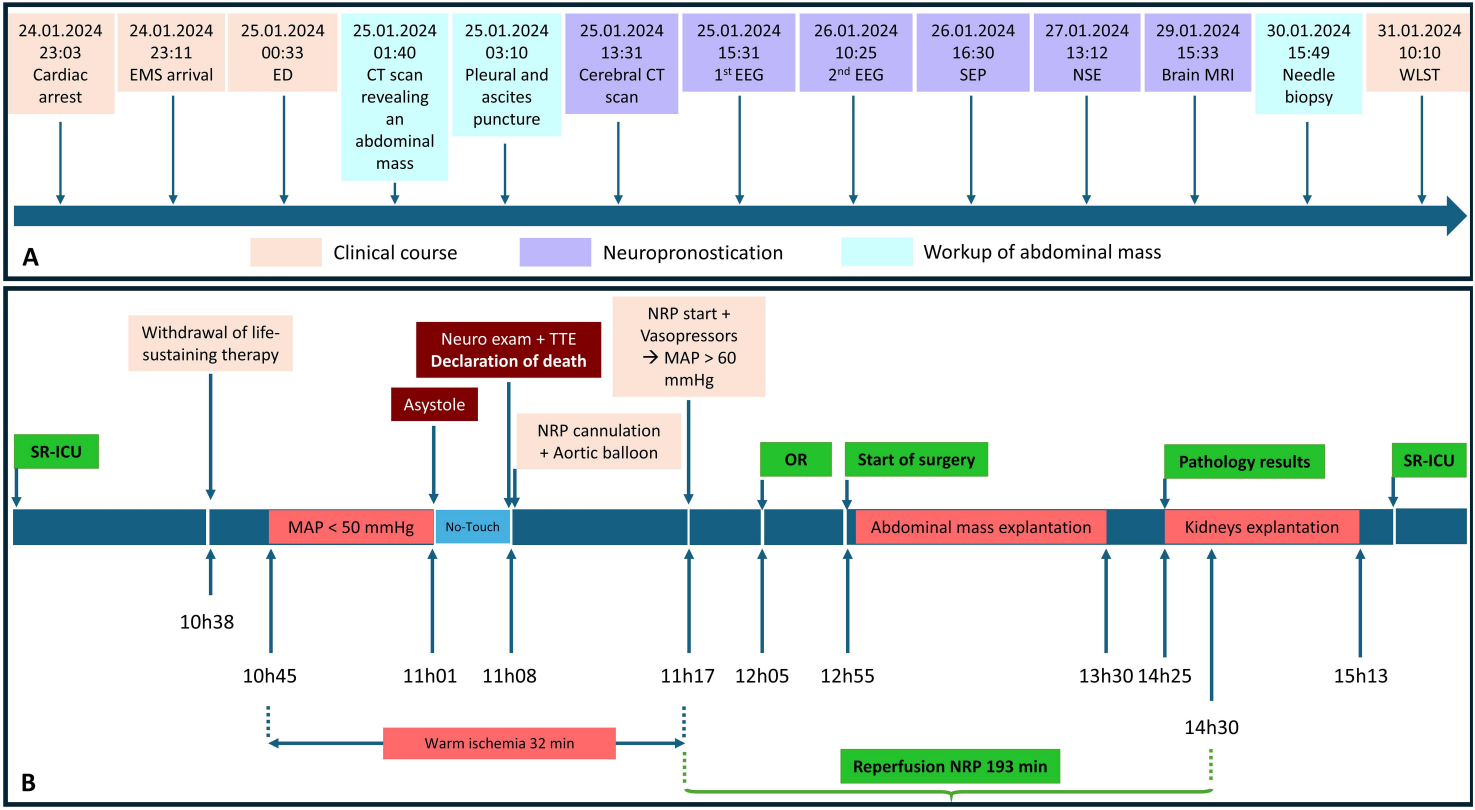
Chronological timeline of donor management, investigations, A-NRP, and surgical procedures. (**A**) Sequence from cardiac arrest, EMS arrival, emergency department admission, diagnostic workup of the abdominal mass (CT scan, pleural/ascites puncture, and needle biopsy), and neuroprognostication (EEG, SEP, NSE, and MRI) until WLST. (**B**) Controlled DCD process with abdominal NRP, showing withdrawal of therapy, decrease in arterial pressure (MAP <50 mmHg), circulatory arrest (asystole), declaration of death with neurological examination and TTE, cannulation and balloon placement, NRP initiation and hemodynamic stabilization, start of surgery (OR), excision of the adnexal mass, pathology results, and kidney procurement. ICU, intensive care unit; EMS, emergency medical service; ED, emergency department; CT, computed tomography; EEG, electroencephalogram; SEP, somatosensory evoked potentials; NSE, neuron-specific enolase; MRI, magnetic resonance imaging; WLST, withdrawal of life-sustaining therapy; MAP, mean arterial pressure; NRP, normothermic regional perfusion; OR, operating room; Neuro, neurological; TTE, transthoracic echocardiography; SR-ICU, single-occupancy ICU room.

Following discussions with the Swiss transplant and surgical teams, a DCD-A-NRP procedure with mass excision and immediate analysis was agreed upon. A schematic pathway summarizing the clinical decision-making process from incidental mass discovery to final organ procurement, including the detailed chronological sequence of withdrawal, NRP, surgery, pathology, and kidney retrieval, is shown in Figure 3. Femoro-femoral cannulation for NRP and placement of the aortic occlusion balloon were feasible despite the pelvic mass and were performed under combined ultrasound and fluoroscopic guidance, without technical difficulty. During A-NRP, blood flow was maintained between 3.5 and 4 L/min at FiO_2_ 50%. No transfusion was required. Donor management included the infusion of 3 L of 0.9% NaCl and 1 100 mL vial of 8.4% sodium bicarbonate to maintain hemodynamic and metabolic stability. Lactate levels decreased (9.3 → 3.6 mmol/L), Aspartate Aminotransferase (AST)/Alanine Aminotransferase (ALT) levels remained stable (48/31 → 43/25 UI/L), and pH normalized, supporting good organ quality. Bilateral oophorectomy was performed under A-NRP, and the frozen section excluded malignancy, with the final pathology confirming an ovarian thecoma. Only the left kidney was successfully transplanted, as the right kidney could not be used due to an acute infection in the intended recipient. The recipient of the left kidney experienced immediate graft function, with urine output of 1,600 mL/24 h, and regained stable renal function at 1 month.

## Discussion

Cancer transmission from deceased donors is rare but potentially fatal (2). Donor cancer acceptance varies depending on recipient characteristics, the expected survival, and cancer type or stage. Despite its rarity, donor-transmitted cancer is difficult to quantify because of reporting bias and limited donor histories (3). Therefore, all donors undergo extensive screening through a review of medical and social history, a physical examination, imaging, and blood tests to exclude contraindications to donation.

Tumor markers CA19-9, CA 125, and CEA are associated with tumor burden and assist in differentiating benign from malignant ovarian masses 6. In our patient, CA 19-9 and CEA levels were low, while CA 125 was elevated. High CA 125 levels are most strongly linked with borderline or malignant tumors, followed by CA 19-9 and CEA 7-9. Moss et al. found that 80% of elevated CA 125 cases were not linked to cancer (4), prompting further investigation in our case. However, CA 125 can also be elevated in benign gynecological or inflammatory conditions, leading to false positives and potential unnecessary donor exclusion. In our donor, this marker profile alone was insufficient to rule out malignancy and was therefore combined with imaging, cytology, and biopsy results. Ultimately, definitive histology under A-NRP was required to safely exclude malignancy before proceeding with organ procurement.

Ovarian aspiration and biopsy can aid diagnosis, but sensitivity is low, and there is a risk of peritoneal tumor spread (5). In our patient, the frozen section showed a bland spindle cell pattern. Because of the size and bilaterality of the tumor, a bilateral oophorectomy was required to exclude malignancy. However, minimizing warm ischemia is vital to preserving graft function.

A-NRP permits oxygenated blood perfusion after death is declared (6). Compared with SRR, A-NRP is safe and improves early transplant outcomes by reducing delayed graft function in DCD kidneys (7, 8). In our patient, A-NRP enabled mass excision and real-time exclusion of malignancy while preserving perfusion, ultimately allowing organ procurement and transplantation. Beyond this patient case, A-NRP may also be valuable for donors presenting with other incidental findings, such as pulmonary or hepatic nodules, where biopsy and rapid pathological assessment could guide procurement decisions. In addition, A-NRP offers additional time for donors with complex surgical histories (e.g., multiple abdominal surgeries or adhesions), who require careful dissection or vascular reconstruction. Such applications could help avoid unnecessary donor exclusion and contribute to expanding the donor pool in centers equipped with A-NRP expertise. Recent experiences from Spain, the United Kingdom, and the United States further support the safety and effectiveness of A-NRP in expanding donor utilization and improving transplant outcomes (7–9).

## Conclusion

In DCD donors with an abdominal mass of uncertain origin, A-NRP enables concurrent organ perfusion and malignancy exclusion, facilitating safe and timely organ procurement and transplantation.

## Data Availability

The original contributions presented in the study are included in the article/Supplementary Material; further inquiries can be directed to the corresponding author.
